# Medical Device Regulation: Time to Improve Performance

**DOI:** 10.1371/journal.pmed.1001277

**Published:** 2012-07-31

**Authors:** Sanket S. Dhruva, Rita F. Redberg

**Affiliations:** 1 University of California, Davis Medical Center, Division of Cardiovascular Medicine, Sacramento, California, United States of America; 2 University of California San Francisco, School of Medicine, Philip R. Lee Institute of Health Policy Studies, San Francisco, California, United States of America

## Abstract

Sanket Dhruva and Rita Redberg comment on a research study evaluating the evidence regarding the performance of device regulatory systems. They argue that adequate funding and increased transparency are vital to future reform.

**Linked Research Article**

This Perspective discusses the following new study published in *PLoS Medicine*:

Kramer DB, Xu S, Kesselheim AS (2012) How Does Medical Device Regulation Perform in the US and EU? A Systematic Review. PLoS Med 9(7): e1001276. doi:10.1371/journal.pmed.1001276

Daniel Kramer and colleagues conduct a systematic review to examine the strengths and weaknesses associated with approaches to medical device regulation in the US and EU.

Medical devices have made a significant contribution to helping patients enjoy longer lives of higher quality. However, numerous weaknesses in their premarket evaluation and post-market surveillance have sometimes left patients worse off from faulty devices. For example, the Sprint Fidelis implantable cardioverter-defibrillator lead (Medtronic, Minneapolis, Minnesota) was approved by the United States Food and Drug Administration (FDA) in 2004. Three years after its approval and subsequent rapid dissemination, this device's high propensity to fracture was finally recognized; first by independent cardiologists and then the FDA and the manufacturer, which voluntarily recalled the device. At least several patient deaths have been attributed to this problem [1],[2]. More than 268,000 patients worldwide have been implanted with this device and have had to choose between risky removal of the defective leads or living with uncertainty of death due to a faulty device. The Sprint Fidelis tragedy exemplifies the need for rigorous and transparent post-market surveillance so that problems with approved devices can be rapidly detected.

## Motivations of Studies of Device Regulation

In this context, the article in this week's *PLoS Medicine* by Daniel Kramer and colleagues is welcomed. They conducted a systematic review of original studies evaluating the performance of device approval and post-market surveillance in the United States and European Union prior to July 2011. They found only 20 studies evaluating the medical device approval process despite an extensive search [3]. Some studies were published in the peer-reviewed literature and suggest that regulatory reforms will be necessary to promote higher quality evidence in studies of the highest-risk devices. These reforms could include increased use of randomization, blinding, and active controls. However, the authors also found a number of non peer-reviewed publications describing the attitudes of industry representatives towards regulatory systems, particularly concern that device approval is not fast enough.

The authors detail a study examining high-risk recalls by the FDA, which found that 78% of medical devices recalled by the FDA over a 5-year period were not considered high risk at the time of approval or were even exempt from review because they were considered so low risk [4]. These data are concerning because they indicate that devices not considered high risk at the time of regulatory evaluation can often subsequently present serious dangers to patients. Alternative non peer-reviewed analyses of the same high-risk recall data conducted by individuals affiliated with device companies assert that 99.5% of submissions did not lead to recalls. This latter study used submissions as the denominator, instead of approved devices [5].

Daniel Kramer and colleagues describe a number of non peer-reviewed studies conducted by device companies that criticize device approval for being too slow [6]. These studies were then critiqued by journal editors in testimony requested by the US Congress; that testimony asserted substantial flaws in the industry-conducted studies [6]. Industry representatives have subsequently cited these self-published reports to argue that any more stringent regulation would be hazardous to patient care by stifling innovation and preventing job creation [7]. More rigorous review for safety and effectiveness may lead to higher pre-approval costs for the device industry. We believe, however, that having sufficient data to assure that a medical device is safe and efficacious is of paramount importance prior to approval with continued post-marketing surveillance to ensure that its benefits outweigh risks in actual use in more patients over time.

## Solutions: Funding, Political Backing, and Transparency

It is a critical time for reform of medical device regulation globally. In Europe, the European Commission is reviewing its process of medical device regulation—one that was established over 20 years ago and has not kept up with technological advances.

In order to conduct rigorous reviews of medical devices and ensure their safety and efficacy prior to approval and in the post-marketing period, it is critical that regulatory agencies have sufficient funding and political backing. The FDA commissioner has stated that the FDA's “resources are outstripped by our responsibilities…there is a continuing need for expansion of investment” [8]. Additionally, the safest and best way to speed review times—as industry prefers because it would reduce costs associated with developing new technologies—is to increase funding for FDA to allow more review staff. The Makower Report, based on a survey of device companies, found frustration with changes in key personnel at the FDA during product evaluation and unpredictability with the agency [9]. Unfortunately, user fees paid by the device industry constitute about one-sixth of the device budget and make the agency financially dependent on the device industry that it is supposed to be regulating [10]. With more resources and financial independence, the FDA would be more likely to retain key personnel and allow them to work closely with companies during the process.

In addition, the FDA requires sufficient political support to fulfill its mission. Unfortunately, instead of recognizing that the FDA is limited in its regulatory ability due to underfunding, some members of Congress—using justification from industry-sponsored studies whose validity has been critiqued [6]—state that the FDA should be penalized for conducting slow device reviews. At a United States House Energy & Commerce meeting last year, Representative Michael Burgess (R-TX), a physician, stated, “It is up to us to provide the funding. It is up to the administrator at the FDA to get the job done with the tools at hand. Why would you pay more for what you are getting? We want more of this? How far away from desirable do we care to be?” [11]. Certainly in the US$350 billion global market for medical devices, additional funding can be obtained for device regulation.

In reforming the device approval process, it is important for all stakeholders in the process to come together. A superb example was the European Society of Cardiology's recent release of a consensus document that suggests several reforms for the outdated European medical device approval process. These include creating a single, coordinated European system to oversee medical device approval and developing product standards [12]. Currently, the European Union's device approval and recall system is said to be limited in usefulness as it lacks transparency and there are few publicly available data [13]. A similar problem has been found with the FDA review process [14]. If regulatory agencies are not adequately equipped to assess devices—and they currently are not—the data should at least be made publicly available to allow for ongoing assessment of devices. Going forward, adequate funding and political backing are essential so that global agencies such as the FDA and EU Notified Bodies can adequately assess medical device safety and efficacy prior to approval and monitor them in the post-marketing period.

## Abbreviations

FDA: United States Food and Drug Administration
